# Long-term persistence with aflibercept therapy among treatment-naïve patients with exudative age-related macular degeneration in a universal health care system: a retrospective study

**DOI:** 10.1186/s12886-022-02593-7

**Published:** 2022-09-19

**Authors:** Reinhard Angermann, Alexander Franchi, Katharina Frede, Victoria Stöckl, Christoph Palme, Martina Kralinger, Claus Zehetner

**Affiliations:** 1 Department of Ophthalmology, Medical University Innsbruck, Anichstraße 35, 6020 Innsbruck, Austria; 2 Department of Ophthalmology, Landesklinikum Mistelbach/Gänserndorf, Lichtensteinstraße 67, 2130 Mistelbach, Austria

**Keywords:** Adherence, Long-term effect of compliance, Loss to follow-up, Persistence, Aflibercept, Age-related macular degeneration

## Abstract

**Background:**

This study  aimed to analyse the persistence rates of treatment-naïve patients with neovascular age-related macular degeneration (nAMD) who received intravitreal aflibercept therapy in a universal health care system.

**Methods:**

In this single-centre retrospective cohort study, we audited data of 918 treatment-naïve patients who received exclusively intravitreal aflibercept therapy for nAMD between September 2015 and May 2021. The primary outcome measures were the rates of treatment nonpersistence (gap in ophthalmological care > 6 months) and long-term nonpersistence (> 12 months).

**Results:**

The rates of nonpersistence and long-term nonpersistence were 12.3% and 3.4% after one year; 22.4% and 9.5% after two years; and 38.3% and 19.3% after five years, respectively. Logistic regression analysis revealed that older age (*p* = 0.045), male sex (*p* = 0.039), requirement for caretakers or ambulance (*p* = 0.001), and low visual acuity of the study eye (*p* = 0.010) or fellow eye (*p* = 0.029) were independent risk factors for long-term nonpersistence. Patients aged > 80 and > 85 years (*p* = 0.013 and *p* = 0.022, respectively) had more than twice the risk for being nonpersistent to therapy within two years of follow-up compared with younger patients. Male patients (*p* = 0.033), patients requiring a caretaker (*p* = 0.038), and patients living > 60 km from the clinic (*p* = 0.029) had a 2 × higher risk of being persistently nonpersistent to therapy.

**Conclusions:**

Patients with nAMD who were treated with aflibercept had lower nonpersistence rates than those reported in current literature. Multiple independent risk factors were correlated with long-term nonpersistence, early nonpersistence, or complete loss to follow-up. Considering the possible consequences of reduced compliance, further strategies are urgently needed for patients at risk of nonpersistence to therapy.

## Background

Exudative age-related macular degeneration (AMD) is a major contributor to vision impairment in elderly patients, accounting for 8.7% of all blindness worldwide [1, 2]. Several anti-vascular endothelial growth factor (VEGF) therapies have been introduced in the last few decades. Depending on the drug, the approved label, and the treatment protocol, monthly to three monthly injections are recommended after an initial loading dose of three injections for three consecutive months [3–6]. However, the number of injections and functional outcomes reported in real-life studies are inconsistent with those reported in randomised clinical trials that are conducted in an idealised and controlled setting [3, 7–10]. This discrepancy in long-term injection frequency and successful preservation of visual acuity in patients with neovascular AMD (nAMD) is most likely due to a lack of adherence to the rigorous treatment and examination regimes. Frequent examination and the timely administration of intravitreal injections are greatly emphasized in the management of patients with nAMD. Nevertheless, little is known about the obstacles that lead to reduced adherence to the treatment regime. Recent studies have reported nonadherence or nonpersistence rates of 32–95% among patients with nAMD [7, 11–13]. However, these studies either had small samples, included non-treatment-naïve patients, or used various anti-VEGF agents in their study cohort. According to a major review by Okada et al. [13], there is little information regarding the compliance rates of patients with nAMD who are treated with aflibercept or other regimens with longer treatment intervals. A previous study identified race and patient income as the main risk factors for loss to follow-up in a healthcare system without general insurance [11]. Since the lack of health insurance was hypothesised as a reason for the underutilization of preventive care and treatment for ophthalmological diseases, the outcomes of this study may not be generalizable to patients in other healthcare systems [14].

In Austria, anti-VEGF therapy is covered by primary public insurance, but only in general hospitals with an ophthalmological department. These special circumstances provide us with the opportunity to assess the risk factors associated with reduced long-term adherence without potential biases related to social status, income, or the ability of patients to receive therapy at a private practice. In the present study, we evaluated the intrinsic and extrinsic risk factors that lead to reduced persistence to therapy among treatment-naïve patients with nAMD. The patients were treated with intravitreal aflibercept on a pro re nata (PRN) regimen at a fixed interval of 8 weeks following an initial loading dose of three injections for three consecutive months (that is, every 4 weeks). The secondary outcome measures were the identification of risk factors leading to early nonpersistence to therapy or complete loss to follow-up (LTFU).

## Methods

### Study population

The data of patients who received anti-VEGF therapy at the Department of Ophthalmology of the University Clinic Innsbruck (Innsbruck, Austria) from September 2015 to May 2021 were retrieved from a structured electronic database and audited retrospectively following a standardised protocol. Approval was received from the Institutional Review Board before the commencement of the research (Medical University Innsbruck, Innsbruck, Austria; approval number 1261/2020). The Review Board waived the requirement for informed consent due to the retrospective nature of the study. All data were anonymized prior to the analysis. The study was conducted in accordance with the tenets of the Declaration of Helsinki.

The inclusion criteria were as follows: (1) patients with nAMD who were treatment-naïve to anti-VEGF therapy and received exclusively aflibercept therapy during follow-up and (2) patients who started with a loading dose of three injections for three consecutive months and were followed up with a fixed therapy interval of aflibercept injections every 8 weeks in a PRN treatment regime.

The exclusion criteria were as follows: patients (1) with diabetes mellitus; (2) with a history of posterior uveitis or retinal vein occlusion; (3) with a history of treatment with other anti-VEGF agents, corticosteroids, or photodynamic therapy; (4) with clinically significant cataract or any other disease that could potentially threaten visual acuity (VA); and (5) who received a follow-up examination at another clinic or private practice during the observation period. In Austria, intravitreal anti-VEGF therapy is covered only by primary public health insurance in hospitals with an ophthalmology department. Therefore, we also excluded patients who lived closer to another clinic with an ophthalmological department.

### Clinical assessment and data collection

In all patients, neovascular AMD was diagnosed at baseline by retinal specialists using fundoscopic examination and fluorescent angiography or optical coherence tomography angiography (OCT-A; Heidelberg Spectralis® OCT, Heidelberg Engineering, Heidelberg, Germany). Patients who were treatment-naïve to anti-VEGF therapy—and had been indicated for intravitreal aflibercept therapy with an initial loading dose of three injections every 4 weeks—were included in the study. At follow-up examinations, additional injections were indicated as triplets of aflibercept every eight weeks on a PRN regime. Appointments for injection and follow-up examinations were routinely scheduled and documented at the end of every consultation.

We collected the following data: demographic data, best-corrected visual acuity (BCVA) at baseline and at every subsequent follow-up visit, number of aflibercept injections, number of follow-up examinations, bilateral or unilateral involvement at baseline, and duration of documented follow-up.

BCVA measurements were recorded as the logarithm of the minimum angle of resolution (logMAR) units. The “distance to the clinic” (that is, the distance of road travel required to travel between the patients’ residence and the clinic) was determined using Google Maps (Google Inc., Mountain View, USA).

### The definition of nonpersistence

Patient persistence was assessed by analysing the occurrence of a gap in ophthalmological care during the given observation period. Based on the consensus criteria described by Okada et al. [15], treatment nonpersistence was defined as an interval of > 6 months without any clinical visits or therapy, and long-term nonpersistence was defined by an interval of > 12 months without any clinical visits or therapy. Early nonpersistence was defined as a gap in ophthalmological care within two years of follow-up; late nonpersistence was defined as a gap in ophthalmological care after two years of follow-up; and complete LTFU was defined as permanent failure to return to treatment in the given observation period. Additionally, we documented the number of nonpersistence episodes and the duration of each nonpersistence episode. Patients who were rescheduled by the clinic/hospital or had themselves actively rescheduled their appointments beyond 6 follow-up months were not considered as nonpersistent.

The primary outcome measures were the prevalence and frequency of nonpersistence and long-term nonpersistence in patients with nAMD receiving intravitreal aflibercept therapy in a primary care centre for retinal diseases in the western region of Austria. The assessment and identification of risk factors associated with nonpersistence to intravitreal aflibercept therapy provided data that were considered as secondary outcomes.

### Statistical analysis

All statistical analyses were performed using SPSS Statistics version 25 (IBM, Armonk, NY, USA).

Demographic data are presented as the number of patients with percentages, normally distributed data as the mean ± standard deviation (SD), and non-normally distributed variables as the median and interquartile range (IQR). The Kolmogorov–Smirnov and Shapiro–Wilk tests were used to determine the normality of distribution for all variables.

We used the chi-squared test and Fisher’s exact test to compare categorical data, the unpaired sample t-test to assess normally distributed data, and the Mann–Whiney U test for non-normally distributed data. Potential risk factors for long-term nonpersistence, early nonpersistence, and complete LTFU were evaluated using a univariate binary logistic regression model. All demographic characteristics that were associated with long-term nonpersistence with a *p*-value of < 0.1 in the univariate analysis were included for multivariate logistic regression. Multivariate logistic regression was used to determine the main risk factors that lead to episodes of nonpersistence, early nonpersistence, and complete LTFU. Binary logistic regression was conducted to determine the odds ratio and 95% confidence interval (CI). Statistical significance was set at a *p*-value of < 0.05.

## Results

### Baseline characteristics of patients and nonpersistence rates

**Fig. 1:**
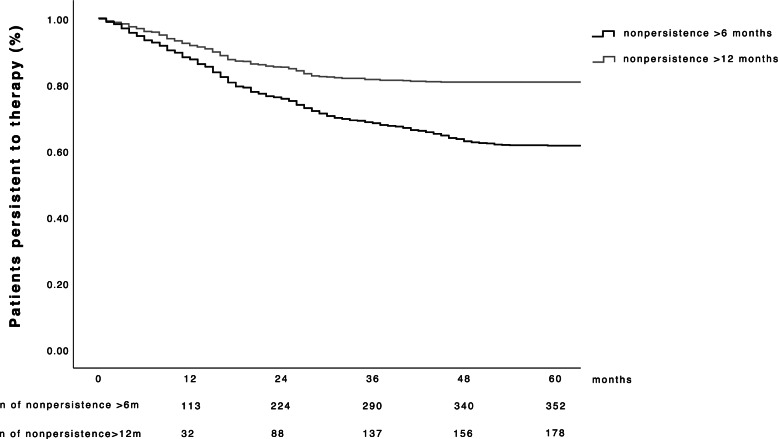
Kaplan–Meier curves. Rates of patient persistence to intravitreal aflibercept therapy are shown over a follow-up period of 60 months

A total of 918 treatment-naïve patients who were treated with intravitreal aflibercept therapy were eligible for analysis. The mean age was 79 ± 7.6 years, and the proportion of female patients in this study cohort was 65% (596/918). The median distance to the clinic was 13.6 (IQR 2.7–38.1) km. In total, 490 (53%) patients arrived independently for therapy, 318 (35%) needed a caretaker, and 110 (12%) arrived in an ambulance. Patients who adhered to their therapy received 10 (IQR 6–16) injections during their follow-up and made a total of 13 (IQR 7–22) visits. In contrast, patients who were nonpersistent to therapy received 7 (IQR 4–11; *p* < 0.001; 99% CI 0.00–0.005) injections and made 9 (IQR 6–16; *p* < 0.001; 99% CI 0.00–0.005) visits during their follow-up. After 5 years, 352 (38.3%) patients were nonpersistent for at least 6 months and 178 (19.4%) for more than 12 months (Fig. 1). The median follow-up period was 31 (IQR 19–48) months for persistent patients, 17 (IQR 12–25) months for the group with nonpersistence, and 15 (IQR 8–25; *p* < 0.001; 99% CI 0.000–0.005) months for the group with long-term nonpersistence. Among the patients who were nonpersistent, 313 (88.9%) had one episode of nonpersistence, 33 (9.4%) had two episodes, and 6 (1.7%) had three episodes. Among the patients who were nonpersistent, 204 (58%) showed nonpersistence and 44 (25%) showed long-term nonpersistence, and all patients returned for further examinations after a median time of 12 (IQR 7–28) months. Moreover, 86 (35%) patients were indicated for further therapy at their return visit. The baseline characteristics of all patients are presented in Table 1.

**Table 1:** Characteristics of patients with neovascular age-related macular degeneration with persistence or long-term nonpersistence to therapy

Baseline characteristics	Persistent group	Long-term nonpersistence group (> 12 months)	*p*-value
N	741 (81)	177 (19)	
Age, years			
≤ 75	214 (29)	42 (24)	< 0.001
76–80	217 (29)	21 (12)	
81–85	165 (22)	45 (25)	
> 85	145 (20)	69 (39)	
Sex			
Male	251 (34)	71 (40)	0.070
Female	490 (66)	106 (60)	
Distance to clinic, km			
≤ 10	335 (45)	77 (43)	0.097
11–30	185 (25)	32 (18)	
31–60	96 (13)	28 (16)	
> 30	125 (17)	40 (23)	
BCVA study eye at baseline, logMAR (SD)	0.62 (0.40)	0.79 (0.45)	< 0.001
BCVA fellow eye at baseline, logMAR (SD)	0.56 (0.50)	0.74 (0.56)	< 0.001
Eye involvement			
bilateral	186 (25)	55 (31)	0.061
unilateral	555 (75)	122 (69)	
Type of transport			
independent arrival	428 (57)	61 (34)	< 0.001
arrival with caretaker	242 (33)	77 (44)	
arrival in an ambulance	71 (10)	39 (22)	

*N* Number, *km* Kilometres, *SD* Standard deviation

### Long-term nonpersistence according to demographic risk factors

Univariate analysis revealed that age, type of transport, sex, distance to the clinic, and bilateral eye involvement (all *p* < 0.08) tended to be associated with an increased rate of long-term nonpersistence (Table 1). Therefore, these demographic factors were included in the multivariate analysis to rule out confounding factors and to identify the key risk factors associated with long-term nonpersistence. Binary logistic regression analysis revealed that the odds of being nonpersistent to therapy were 64% higher among patients aged > 85 years than among patients aged ≤ 75 years (*p* = 0.045, Table 2). Male patients had a 45% higher risk of being nonpersistent than female patients (*p* = 0.039). Additionally, patients requiring a caretaker or patients arriving in an ambulance had a 2 × and 3 × higher chance of being nonpersistent for > 12 months (*p* = 0.001 and *p* < 0.001, respectively).

**Table 2:** Risk factors for long-term nonpersistence among patients with age-related macular degeneration

	Long-term nonpersistence (> 12 months)	Univariate model *p*-value (odds ratio; 95% CI)	Multivariate model^+^ *p*-value (odds ratio; 95% CI)
N	177 (19.2)		
Age, years			
≤ 75	42 (16.4)	Reference	Reference
76–80	21 (8.8)	0.013^*^ (2.02; 1.16–3.53	0.001^*^ (2.56; 1.44–4.54)
81–85	45 (21.4)	0.167 (1.39; 0.87–2.22)	0.700 (1.10; 0.67–1.79)
> 85	69 (32.2)	< 0.001^*^ (2.43; 1.56–3.76)	0.045^*^ (1.64; 1.01–2.65)
Sex			
Male	71 (22.0)	Reference	Reference
Female	106 (17.8)	0.119 (1.31; 0.55–1.07)	0.039^*^ (1.45; 1.02–2.08)
Distance to clinic, km			
≤ 10	77 (18.9)	Reference	Reference
11–30	32 (14.7)	0.188 (0.74; 0.47–1.16)	0.249 (1.32; 0.65–2.08)
31–60	28 (22.6)	0.377 (1.25; 0.76–2.03)	0.025^*^ (2.04; 0.65–2.32)
> 60	40 (24.2)	0.158 (1.37; 0.87–2.11)	0.191 (1.36; 0.86–2.15)
Eye involvement			
bilateral	55 (23.0)	Reference	Reference
unilateral	122 (18.1)	0.113 (1.352; 0.94–1.94)	0.536 (1.13; 0.77–1.65)
Type of transport			
independent arrival	61 (34)	Reference	Reference
arrival with caretaker	77 (44)	< 0.001^*^ (2.23; 1.54–3.23)	0.001^*^ (1.99; 1.33–2.95)
arrival in an ambulance	39 (22)	< 0.001^*^ (3.83; 2.39–6.16)	< 0.001^*^ (3.1; 1.84–5.25)

*CI* Confidence interval, *N* Number, *km* Kilometres

^*^ indicates *p*-value < 0.05

^+^ binary logistic regression adjusted for age, sex, and type of transport

### Long-term nonpersistence according to visual acuity

Binary logistic regression adjusted for age, sex, distance to the clinic, and type of transport revealed that patients with a low VA (> 1.0 logMAR) of the study eye at baseline had an 86% greater chance of being nonpersistent to therapy than patients with a good VA (< 0.4 logMAR) of the study eye at baseline (*p* = 0.010). Similarly, a low VA of the fellow eye at baseline correlated with a higher rate of nonpersistence than did a good VA of the fellow eye at baseline (*p* = 0.029) (Table 3).

**Table 3:** Long-term nonpersistence according to visual acuity among patients with age-related macular degeneration

	Long-term nonpersistence (> 12 months)	Univariate model *p*-value (odds ratio; 95% CI)	Multivariate model^+^ *p*-value (odds ratio; 95% CI)
Visual acuity, logMAR			
Study eye at baseline			
≤ 0.4	32 (14.5)	Reference	Reference
0.4–1.0	64 (15.8)	0.561 (1.15; 0.72–1.82)	0.972 (1.01; 0.63–1.62)
> 1.0	81 (27.9)	< 0.001^*^ (2.36; .1.49–3.74)	0.010^*^ (1.86; 1.16–2.99)
Fellow eye at baseline			
≤ 0.4	61 (15.2)	Reference	Reference
0.4–1.0	38 (17.9)	0.379 (1.22; 0.78–1.90)	0.025 (1.57; 1.05–2.33)
> 1.0	78 (26.4)	< 0.001^*^ (2.01; .1.38–2.93)	0.029^*^ (1.65; 1.05–2.58)
Study eye before episode of nonpersistence			
≤ 0.4	37 (15.7)	Reference	Reference
0.4–1.0	45 (7.2)	0.934 (1.02; 0.64–1.64)	0.176 (0.74; 0.47–1.15)
> 1.0	95 (22.7)	0.015^*^ (1.68; 1.11–2.56)	0.08 (0.70; 0.46–1.04)
Fellow eye before episode of nonpersistence			
≤ 0.4	47 (29.2)	Reference	Reference
0.4–1.0	45 (8.4)	0.084 (1.49; 0.95–2.33)	0.406 (1.21; 076–1.93)
> 1.0	85 (40.1)	0.003^*^ (1.81; 1.22–2.68)	0.069 (1.46; 0.97–2.2)
Study eye–change in visual acuity until episode of nonpersistence			
≤ -0.2	69 (19.1)	Reference	Reference
- 0.2 to 0.2	61 (20.1)	0.792 (1.05; 0.72 – 1.55)	0.992 (1.00; 0.67–1.50)
> 0.2	47 (19.3)	0.931 (1.01; 0.67 – 1.54)	0.919 (0.98; 0.64–1.50)

*CI* Confidence interval, *logMAR* Logarithm of minimum angle of resolution

+ binary logistic regression adjusted for age, sex, and type of transport

^*^ indicates *p*-value < 0.05

### Characterisation of patients with early nonpersistence and of patients who returned for therapy

To analyse the risk factors for early nonpersistence, we divided patients into two subgroups: the early nonpersistence group (nonpersistence within two years of follow-up) and the late nonpersistence group (nonpersistence after two years of follow-up). Binary logistic regression analysis of these groups revealed that patients aged 81–85 years and > 85 years were at more than twice the risk of developing early nonpersistence (*p* = 0.013 and *p* = 0.022, respectively) than did younger patients (see Table 4). Compared with patients with a good VA of their study or fellow eye, patients with either a moderate VA (0.4–1.0 logMAR; *p* = 0.364 and *p* = 0.255, respectively) or a low VA (*p* = 0.352 and *p* = 0.070, respectively) did not show a high rate of early nonpersistence.

**Table 4:** Risk factors of early nonpersistence among patients with age-related macular degeneration

	Early nonpersistence	Late nonpersistence	Univariate model *p*-value (odds ratio; 95% CI)	Multivariate model^+^ *p*-value (odds ratio; 95% CI)
N	219	133		
Age, years				
≤ 75	43 (12)	45 (13)	Reference	Reference
76–80	41 (12)	26 (7)	0.128 (1.65; 0.87–3.14)	0.227 (1.51; 0.77–2.93)
81–85	60 (17)	27 (8)	0.007^*^ (2.32; 1.25–4.31)	0.013^*^ (2.27; 1.20–4.34)
> 85	75 (21)	35 (10)	0.006^*^ (2.24; 1.25–4.00)	0.022^*^ (2.07; 1.12–3.86)
Sex				
Male	85 (24)	43 (12)	Reference	Reference
Female	134 (38)	90 (26)	0.221 (0.75; 0.478–1.19)	0.118 (0.68; 0.42–1.10)
Distance to clinic, km				
≤ 10	96 (27)	61 (17)	Reference	Reference
11–30	50 (14)	22 (6)	0.214 (1.46; 0.80- 2.64)	0.183 (1.52; 0.82–2.79)
31–60	30 (9)	27 (8)	0.279 (0.71; 0.39–1.31)	0.284 (0.71; 0.38–1.33)
> 60	43 (12)	23 (7)	0.550 (1.20; 0.66–2.19)	0.522 (1.23; 0.65–2.30)
Type of transport				
independent arrival	92 (26)	65 (18)	Reference	Reference
arrival with caretaker	83 (24)	55 (16)	0.787 (1.07; 0.67–1.70)	0.739 (0.92; 0.56–1.51)
arrival in an ambulance	44 (12)	13 (4)	0.014^*^ (2.39; 1.19–4.79)	0.061 (2.05; 0.97–4.33)

*CI* Confidence interval, *N* Number, *km* Kilometres

^+^ binary logistic regression adjusted to age and type of transport

^*^ indicates *p*-value < 0.05

We also subdivided patients who were nonpersistent into two groups: patients who returned to therapy (return group) and patients with complete loss to follow-up (LTFU group). Multivariate analysis adjusted for age, sex, distance to the clinic, and type of transport showed that male patients had a 67% higher chance of being completely LTFU than did female patients (*p* = 0.033). Additionally, patients staying at a distance of > 60 km from the clinic and patients who needed an ambulance to arrive at the clinic had a 2 × higher risk of being completely LTFU than did patients who lived in proximity to the clinic (*p* = 0.029 and *p* = 0.038, respectively) (see Table 5). Moderate VA (*p* = 0.671) or low VA (*p* = 0.150) of the study eye did not correlate with high rates of return to therapy after nonpersistence. In contrast, patients with a low VA of the fellow eye had a 216% higher chance of being completely LTFU than did patients with a good VA of the fellow eye (*p* = 0.003; 95% CI 1.30–3.62).

**Table 5:** Risk factors for complete loss to follow-up among patients with age-related macular degeneration

	Complete loss to follow-up	Return after nonpersistence	Univariate model *p*-value (odds ratio; 95% CI)	Multivariate model^+^ *p*-value (odds ratio; 95% CI)
N	148	204		
Age, years				
≤ 75	34 (10)	54 (15)	Reference	Reference
76–80	18 (5)	49 (14)	0.126 (1.71; 0.86–3.42)	0.068 (1.96; 0.95–4.02)
81–85	40 (11)	47 (14)	0.326 (0.74; 0.41–1.35)	0.486 (0.798; 0.42–1.50)
> 85	56 (16)	54 (15)	0.086 (0.61; 0.344–1.07)	0.281 (0.71; 0.38–1.32)
Sex				
Male	61 (17)	67 (19)	Reference	Reference
Female	87 (25)	137 (39)	0.108 (1.43; 0.925–2.23)	0.033^*^ (1.66; 1.04–2.64)
Distance to clinic, km				
≤ 10	59 (17)	98 (28)	Reference	Reference
11–30	26 (7)	46 (13)	0.804 (1.08; 0.60–1.92)	0.859 (1.05; 0.58–1.92)
31–60	27 (8)	30 (8.5)	0.210 (0.68; 0.37–1.25)	0.155 (0.63; 0.33–1.91)
> 60	36 (10)	30 (8.5)	0.022^*^ (0.51; 0.28–0.91)	0.029^*^ (1.98; 1.07–3.64)
Type of transport				
independent arrival	53 (15)	104 (30)	Reference	Reference
arrival with caretaker	64 (18)	74 (21)	0.028^*^ (1.69; 1.06–2.72)	0.164 (0.70; 0.42–1.16)
arrival in an ambulance	31 (9)	26 (7)	0.014^*^ (2.34; 1.26–4.32)	0.038^*^ (2.05; 1.03–4.04)

*CI* Confidence interval, *N* Number, *km* Kilometres

^*+*^ binary logistic regression adjusted for distance and type of transport

^*^ indicates *p*−value < 0.05

## Discussion

Nonpersistence and nonadherence to anti-VEGF therapy is one of the biggest challenges in the management of patients with nAMD. These are major contributing factors to the rather sobering results of real-life studies, which differ considerably from the outcomes of major clinical trials [3, 7–10]. The complex and multifactorial reasons associated with nonadherence and reduced compliance to treatment regimens among patients with nAMD were explored in a major review by Okada et al. [13]. In this review, the authors reported that the rates of nonadherence varied from 32 to 95% across studies, depending on the definition criteria used in different studies.

In our study, we analysed nonadherence to intravitreal aflibercept therapy in treatment-naïve patients with nAMD. The rates of nonpersistence for 6 and 12 months were 12.3% and 3.4% after one year and 22.4% and 9.5% after two years, respectively. In contrast, a cohort study conducted by Obeid et al. [11] in the tristate area of Pennsylvania, New Jersey, and Delaware revealed an LTFU of 12 months in 22% of cases at a median follow-up time of 2.4 years. Unlike Austria, which adopts a primarily public healthcare system, the United States does not provide a universal healthcare program. Consequently, anti-VEGF therapy is not covered. The impact of insurance status on patient adherence is perfectly reflected in the report by Obeid et al. [11], which identified the income and ethnicity of patients as major contributors to their reduced compliance. Another retrospective study analysed the data of 201 patients receiving ranibizumab therapy at 4-week intervals in France, which also provides universal health care. The study reported LTFU episodes of at least 6 months in 26% of cases after one year, 38% of cases after two years, and > 50% of cases within 4 years [7]. In contrast, the results of our study revealed a 38% rate of nonpersistence for 6 months in exclusively treatment-naïve patients receiving aflibercept treatment at 8-week intervals over a follow-up period of 5 years.

The results of a phone survey revealed that the burden of follow-up visits contributes to nonadherence to intravitreal therapy in one out of four cases [7]. The same study reported that the distance to the clinic was the main reason for therapy break-off in every second patient. In this study, we found that patients who had to travel more than 30 km to reach the clinic were predisposed to be nonpersistent to therapy. Similarly, patients travelling more than 60 km were at twice the risk of being completely LTFU. Older age was also a major contributor to nonadherence and was associated with increased risk of long-term nonpersistence or early nonpersistence by a factor of 1.5–2.0. Compared with female patients, male patients showed a high rate of long-term nonpersistence and high proportion of complete LTFU. Comorbidities and morbidity are known to increase with age, and these factors impede the ability of patients to operate independently [16]. Patients in need of anti-VEGF therapy must adhere to numerous follow-ups and treatment schedules, and such dependencies can be devastating to their ability to perform follow-up visits. The importance of patients’ ability to operate independently is highlighted by the finding that long-term nonpersistence increased by up to 36% among patients who needed a caretaker or an ambulance. This proportion was significantly higher than that among patients who were able to travel independently. Moreover, the proportion of patients who returned after being nonpersistent was significantly higher in the group that travelled independently than in the group that required an ambulance. Thus, the lack of mobility was considered an independent risk factor, as it doubled or tripled the odds of long-term nonpersistence and complete LTFU among our cohort.

A notable finding of the present study was the correlation between poor VA of the study/fellow eye at baseline and an increased rate of long-term nonpersistence. Our results suggest that patients had twice the risk of being completely LTFU if their fellow eye had a poor VA at baseline. Polat et al. reported that a disbelief in treatment benefit and fear of injection were the most frequent reasons for treatment discontinuation [17]. The equivocal results of other studies regarding the association between noncompliance and VA suggest that the role of vision in noncompliance has yet not been sufficiently explored. The VA of the fellow eye, which was taken into account in this study, was found to be an important factor contributing to nonpersistence. However, most studies do not document the bilateral VA and do not clarify whether the patients were treatment-naïve [7, 11, 12].

This study has some limitations. First, due to its retrospective nature, we could not obtain information regarding person-specific reasons for nonpersistence to therapy. Therefore, we were unable to address the needs of individual patients in order to improve their adherence to therapy. A larger-scale retrospective phone survey is needed that stratifies the responses according to person-specific reasons for nonpersistence and treatment quality. Such analyses can help assess the special needs of patients in the management of nAMD. Importantly, approximately 18% of nonpersistence for 6 months occurred during the corona virus disease 2019 (COVID-19) pandemic. This is of great concern because if left untreated, a potentially irreversible deterioration of macular function can occur relatively quickly in patients with nAMD [18]. The impact of COVID-19 pandemic-related restrictions on the outcomes of nAMD patients has been reported in a previous study by our research team [19]. Moreover, other studies have also reported a decline in VA following a gap in ophthalmological care, especially in eyes with nAMD [20, 21].

The strengths of the present study are its considerably large sample size of treatment-naïve nAMD patients who received exclusively intravitreal aflibercept at one tertiary centre. As mentioned above, general insurance in Austria only covers anti-VEGF therapy in public hospitals with an ophthalmological department. Therefore, we were able to rule out the bias introduced by patient income and the potential bias introduced by patients receiving therapy from other ophthalmologists. This is a unique feature of the present study. Furthermore, we were able to characterise patients who returned for therapy, providing new insights into the complex factors associated with nonadherence to anti-VEGF therapy in the real world.

Novel anti-VEGF drugs with longer durability are being developed at present. Nevertheless, other strategies—such as reminder softwares or teaching programs—may be needed to ensure better adherence to the rigid therapy programs currently in place for nAMD, thereby reducing the episodes of nonpersistence [22]. However, these strategies may be difficult to apply for older patients, and alternative routes in the growing fields of telemedicine and artificial intelligence may offer viable options [23–26]. Additionally, strategies that reduce the number of visits may be beneficial in a pandemic situation.

## Conclusions

Compared with studies reporting nonadherence to other anti-VEGF agents, the present study revealed a low nonpersistence rate among patients with nAMD who were being treated with intravitreal aflibercept. A lack of mobility doubled or tripled the risk of long-term nonpersistence and complete LTFU. Other key risk factors for early nonpersistence, long-term nonpersistence, or complete LTFU were older age, male sex, long distances to the clinic, and low VA at baseline. The higher nonpersistence rates of patients who had to travel longer distances to the clinic and of patients who required a caretaker or ambulance may indicate structural problems in the quality of care offered to patients with nAMD. There is an urgent need for novel strategies aimed at preserving visual function in patients at risk of nonpersistence.

## Data Availability

The data of this research will be available on reasonable request from the corresponding author.

## Abbreviations

VEGF: Vascular endothelial growth factor
AMD: Age-related macular degeneration
nAMD: Neovascular AMD
LTFU: Loss to follow-up
PRN: Pro re nata
OCT-A: Optical coherence tomography angiography
VA: Visual acuity
BCVA: Best-corrected visual acuity
logMAR: Logarithm of the minimum angle of resolution
SD: Standard deviation
IQR: Interquartile range
CI: Confidence interval
COVID-19: Corona virus disease 2019
